# Identification of Transcriptional Variation in Aortic Remodeling Using a Murine Transverse Aortic Constriction (TAC) Model

**DOI:** 10.3389/fcvm.2020.581362

**Published:** 2020-11-12

**Authors:** Xinlu Zhang, Ting Feng, Xin-Xin I. Zeng, Hongbin Liang, Bo Situ, Qiuxia Zhang, Fengyun Zhou, Yejia Chen, Tao Wang, Du Cai, Xinxin Lin, Jiancheng Xiu, Lei Zheng

**Affiliations:** ^1^Department of Laboratory Medicine, Nanfang Hospital, Southern Medical University, Guangzhou, China; ^2^Department of Cardiology, Nanfang Hospital, Southern Medical University, Guangzhou, China; ^3^Human Genetics Program, Sanford Burnham Prebys Medical Discovery Institute, La Jolla, CA, United States

**Keywords:** hypertension, arterial remodeling, transverse aortic constriction (TAC), transcriptional changes, bioinformatics analysis

## Abstract

Arterial remodeling is a major pathological consequence of hypertension, which is recognized as the most common chronic non-communicable disease. However, the detailed mechanism of how arterial remodeling is induced by hypertension has not yet been fully elucidated. Evaluating the transcriptional changes in arterial tissue in response to elevated blood pressure at an early stage may provide new insights and identify novel therapeutic candidates in preventing arterial remodeling. Here, we used the ascending aorta of the transverse aortic constriction (TAC) model to induce arterial remodeling in C57BL/6 male mice. Age-matched mice were subjected to sham surgery as controls. The TAC model was only considered successful if the mice conformed to the criteria (RC/LC blood flow velocity with 5–10-fold change) 1 week after the surgery. Two weeks after surgery, the ascending aorta developed severe remodeling in TAC mice as compared to the sham group. High throughput sequencing was then applied to identify differentially expressed (DE) transcripts. In silicon analysis were then performed to systematically network transcriptional changes. A total of 1,019 mRNAs were significantly changed between TAC and the sham group at the transcriptional level. GO (Gene Ontology) and KEGG (Kyoto Encyclopedia of Genes and Genomes) analysis revealed that stress/stimulus/immune-related biological processes played a crucial role during arterial remodeling. Our data provide a comprehensive understanding of global gene expression changes in the TAC model, which suggests that targeting inflammation and vascular smooth cell transformation are potential therapeutic strategies to interfere with the aortic remodeling at an early stage in the development of hypertension.

## Introduction

Hypertension is the most prevalent chronic non-infectious disease and one of the most significant risk factors for cardiovascular and cerebrovascular diseases (1, 2). The World Health Organization estimates that more than 1.13 billion people worldwide suffer from high blood pressure. More importantly, due to the aging population, the number of hypertension patients is expected to be over 1.5 billion by 2025 globally (3). The increasing burden of hypertension not only enhances the morbidity and mortality of cardiovascular and kidney diseases, it also causes increasing economic costs that affect the whole society (4). Although clinical anti-hypertensive drugs, including ACEI, ARB, and β-blockers, have certain functions in inhibiting vascular remodeling while also reducing blood pressure, the clinical benefits of these drugs are not satisfactory (5). Therefore, there is high demand and an urgent need to uncover new therapeutic targets for hypertension therapies.

Arterial remodeling is one of the significant pathological consequences of high blood pressure, which ultimately induces arteriosclerosis and tissue damage in various organs (6, 7). Arterial remodeling is influenced by hemodynamic loading, neuro-hormonal activation, inflammation, immunity, and other potential factors. Among them, hemodynamic changes can activate vascular biochemical signals via sensing mechanical transduction variation, followed by multiple cytological pathological and physiological alterations, such as cell hypertrophy, proliferation, apoptosis, and extracellular matrix (ECM) turnover. The classical phenotypic transformation of arterial remodeling is located in the median vascular smooth muscle cells, which is manifested as hypertrophy, high proliferation, increased migration to the endothelium, and a large amount of ECM deposition (8, 9). The ultimate goal of inhibiting hypertension-induced vascular remodeling is to prevent end-organ damage, whereas inflammation is a key mediator between these two events (10), thus, targeting inflammation is a straightforward strategy to avoid hypertension associated organ damage.

The TAC-induced hypertension model is a well-established system that accurately mimics pathological changes, including thickening of the medial and adventitia of blood vessels as well as inhibition of endothelial cells-vascular smooth muscle cells (EC-VSMC) dependent vasomotor function (11, 12). Although AT1 receptor activation plays an important role in pressure-induced arterial remodeling in the TAC model (13), the sophisticated mechanism of how hypertension induces arterial remodeling remains unclear. Here, we used RNA Sequencing (RNA-seq) to identify DE transcripts during the arterial remodeling development and to validate these transcriptional alternations by analyzing mouse aorta tissues with multiple assays, which can reveal novel biomarkers and therapeutic targets for hypertension-induced arterial remodeling at an early stage of the disease.

## Materials and Methods

### Establishment of Transverse Aortic Constriction (TAC) Model

All animal care and experimental procedures were approved by the Institutional Animal Care and Use Committee of Southern Medical University and performed in accordance with the Guide for the Care and Use of Laboratory Animals published by the United States National Institutes of Health. C57BL/6J male mice (8 weeks old, 20–25 g) were intraperitoneally anesthetized with a mixture of xylazine (5 mg/kg) and ketamine (100 mg/kg), and awareness was monitored by the negative tail pinch reflex. Mice were fixed on a temperature-controlled operating table in the supine position. After an endotracheal tube was inserted, the mice were supported by a mouse mini-ventilator (Alcott biotech, Shanghai, China). The respiration rate was set to 150 breaths/min, tidal volumes to 2 ml/min, and inspiration/expiration ratio to 1:1.3. After depilation with depilatory cream and routine disinfection, the mouse skin was incised next to the sternum to the second rib. By penetrating the second intercostal space with forceps to expose the thymus, and then displace the thymus until the aortic arch appeared clearly. A curved needle was used to assist in pulling the 6-0 suture under the aortic arch. A27G needle was specially processed as a spacer for TAC-ligation and the needle was withdrawn after tight banding. Finally, the chest was closed and the animal was monitored until fully recovered. Sham-operated mice were subjected to the same procedure but without banding. One week after the operation, mice were subject to ultrasound to examine whether the TAC model was successfully established according to previously reported standards (14, 15). Two weeks later, mice were sacrificed by anesthesia overdosing with intra-peritoneal injection of pentobarbital sodium and cervical dislocation, and their ascending aortas were collected for further analysis. For histological examinations, aortas were fixed in 4% formalin, whereas for other analyses the aortas were stored at −80°C until the analysis.

### Invasive Measurement of Hemodynamics

Left ventricular (LV) hemodynamics were evaluated before the animals were sacrificed. LV hemodynamics were performed under anesthesia (1.5–2% isofluorane, 2 L/min oxygen flow rate) with mice incubated and ventilated. A 1.0 F catheter (Millar Instruments, Inc., Houston, TX) was inserted into the right carotid artery and advanced to the LV. The parameters of the LV systolic pressure (LVSP), LV end-diastolic pressure (LVEDP), and the maximum and minimum rates of change of the LV pressure (max dp/dt and min dp/dt, respectively) were recorded. The exponential time constant of LV relaxation (τ) was then calculated using Power Lab software (blood pressure module; AD Instruments, Shanghai Trading Co., Shanghai, China).

### Echocardiography

Echocardiography was performed in anesthetized (3–5% isoflurane for induction, 1–1.5% for maintenance, with a 0.8 L/min oxygen flow rate) mice at 7 and 14 days after surgery using a VEVO2100 Imaging System (Visual Sonics, ON, Canada) with a 40-MHz probe. After the mice were fixed on a heating plate (37°C), the throat and the chest of mice were depilated, followed by ultrasonic coupling agent treatment. Firstly, the aorta was located in B-Mode, with the presence of the brachiocephalic trunk, the left common carotid artery, and the left subclavian artery as the standard view. After successful positioning and switching to color Doppler Mode, the ligation stenosis was selected as the measurement point, then switched to pulsed-wave Doppler Mode, images were acquired with cine store and frame store for subsequent measurements and calculations. Meanwhile, the probe was placed at an angle of 45 degrees on each side of the neck to assess blood flow velocities in the right common carotid artery (RCCA) and the left common carotid artery (LCCA), respectively. Subsequently, the long axis and short axis of the heart were acquired in B-Mode, while the measurements were performed under M-Mode for evaluation of cardiac function. All scanning and analysis were performed in a double-blind fashion. Only mice with a right carotid/left carotid (RC/LC) flow ratio within a certain range (5–10) in the TAC group and a sham animal with RC/LC of ~1 1 week after surgery were included in further analyses (13).

### Histomorphometric Study

Two weeks after the procedure, the animals were anesthetized and fixed with 20 ml of 1 × PBS perfusion, followed by 10 ml of 4% paraformaldehyde for 3 min under physiological pressure. The ascending aorta was then excised and further fixed overnight. The tissues were then embedded transversely in paraffin and cut at 4 μm thickness. Cross-sections were stained by hematoxylin and eosin (H&E) (Biosharp, Hefei, China) according to standard protocol. For morphometric analysis, images of H&E stained cross-sections were acquired and recorded with the Olympus BX53® microscope (Center Valley, PA, USA) at 400 × magnifications. The thickness of vascular media was analyzed using ImageJ software (National Institutes of Health, Bethesda, MD). The medial area was defined as the area between the external and internal elastic lamina. The adventitia area was defined as the area between the external elastic lamina and the tunica externa as well as the outermost layer of the vessel. Sections were also stained with Sirius red (Leagene, Beijing, China) to determine the collagen content and with improved Weigert's staining (Leagene, Beijing, China) to detect the expression of elastin. Vascular morphology was determined using an Olympus cell Sens imaging system at 400 × magnification. The expression of elastic fibers was calculated as the area of elastic fibers/area of the media × 100%, and the area of collagen fibers was directly used to show the expression of collagen fibers. We used ImageJ software for all these area measurements, and two sections from one specimen derived from each mouse were selected randomly. Five mice for each group were used in the final analysis.

### Immunohistochemistry and Immunofluorescence

Mouse tissues were de-paraffinized, rehydrated, and incubated in 3% hydrogenperoxide for 10 min to block endogenous peroxidase activity. The sections were placed into a pre-boiled citric acid buffer solution for antigen retrieval using routine methods. Next, slides were cooled down and then washed 3 times in 1 × PBS. Sections were then pre-incubated with 5% bovine serum to block non-specific binding for 1 h at room temperature and then incubated with primary antibodies at 4°C overnight. Immunohistochemistry staining was performed with a two-step detection kit (Zhongshan Golden Bridge Biotechnology, Beijing, China) according to the manufacturer's protocol. After three washes with 1 × PBS, the slides were exposed to diaminobenzidine with a DAB IHC Detection Kit (Zhongshan Golden Bridge Biotechnology, Beijing, China) for positive immunostaining and counterstained with Mayer's hematoxylin (Solarbio, Beijing, China). Images were captured with an Olympus BX53® microscope (Center Valley, PA, USA) at 400 × magnifications. We used positive staining to establish proteins of interest, which were evaluated using average optical density (AOD) across the section area. The primary antibodies were α-smooth muscle actin rabbit mAb (α-SMA)(1:700, CST, USA), rabbit anti-collagen I polyclonal antibody(COL-I)(1:500, Bioss, Beijing, China), Angptl1rabbit mAb (1:500, Santa Cruz, USA) and PCNA rabbit mAb (1:8,000, CST, USA), and rabbit anti-TGF-β1 polyclonal antibody (1:500, Bioss, Beijing, China). ImageJ software was used (8–10 randomly chosen sections per sample) for quantification. For immunofluorescence staining, most of the procedures were protected from light. Goat Anti-Rabbit IgG(H+L) Cy3 (1:500, Bioworld, USA) secondary antibodies were added for 1 h at 37°C. Slides were then stained with DAPI (Bestbio, Shanghai, China) according to the manufacturer's instructions. Elastic fibers were monitored with its auto-fluorescence. The images were obtained with a Leica (TCS Sp8) confocal microscopy (Leica, Germany) at 400 × magnification. The primary antibodies used in this study were PCNA rabbit mAb (1:800, CST, USA) and rabbit anti-TGF-β1 polyclonal antibody (1:200, Bioss, Beijing, China).

### Western Blotting

Proteins were extracted from the ascending aorta tissues by RIPA buffer (FDbio, Hangzhou, China) which were added with protease inhibitor (Solarbio, Beijing, China) (1:100) and quantified by BCA assay (Thermo Fisher, USA). Protein samples were mixed with a loading buffer and PageRuler™ Prestained Protein Ladder (10–180 kDa) (Thermo Fisher, USA) protein marker and separated by 10% SDS-PAGE, then transferred onto Immobilon-PSQ PVDF Membrane (Millipore, USA). The membranes were incubated at room temperature for 1 h in blocking buffer (5% non-fat powder in Tris-buffered saline and Tween 20 (TBST) buffer). After blocking, the membranes were incubated with primary antibodies overnight at 4°C. The primary antibodies we used were α-SMA rabbit mAb (1:700, CST, USA), rabbit anti-COL-I polyclonal antibody (1:500, Bioss, Beijing, China), Angptl1mouse mAb (1:500, Santa Cruz, USA), PCNA rabbit mAb (1:8,000, CST, USA), rabbit anti-TGF-β1 polyclonal antibody (1:500, Bioss, Beijing, China), GAPDH mouse mAb (1:5,000, Proteintech, USA), and β-Tubulin mouse mAb (1:50,000, Proteintech, USA). After washing 3 times with TBST, they were incubated with goat anti-rabbit IgG-HRP (1:5,000, FDbio, Hangzhou, China) or goat anti-mouse IgG-HRP (1:5,000, FDbio, Hangzhou, China). After another 3 washes with TBST, immunoreactive bands were detected by ECL Substrate (FDbio, Hangzhou, China) and visualized by the chemiluminescence imager GeneGnome XRQ (Syngene, MD). To calculate the relative expression, the intensity of α-SMA, COL-I, and TGF-β1 were normalized to GAPDH, while PCNA were normalized to β-Tubulin using ImageJ.

### RNA Sequencing

Total RNA was extracted using the Trizol reagent kit (Invitrogen, Carlsbad, CA, USA) according to the manufacturer's protocol (*n* = 4 per group), 2 weeks after the TAC surgery. RNA quality was assessed on an Agilent 2100 Bioanalyzer (RIN>7) (Agilent Technologies, Palo Alto, CA, USA) and double-checked using RNase free agarose gel electrophoresis. After total RNA was extracted, 1 μg of total RNA was used to prepare the mRNA library. The enriched mRNAs were fragmented into short fragments using a fragmentation buffer and reverse transcribed into cDNA with random primers. Second-strand cDNA were synthesized by DNA polymerase I, RNase H, dNTP (dUTP instead of dTTP). Next, the cDNA fragments were purified with QiaQuick PCR extraction kit(Qiagen, Venlo, The Netherlands), end-repaired, poly(A)-added, and ligated to Illumina sequencing adapters. Then UNG (Uracil-N-Glycosylase) was used to digest the second-strand cDNA. The digested products were size selected by agarose gel electrophoresis, PCR amplified, and sequenced using Illumina HiSeq™ 4000 by Gene De novo Biotechnology Co. (Guangzhou, China). To obtain high-quality clean reads, reads were further filtered by fastp (16) (version 0.18.0). The criteria were as follows: (1) removing reads containing adapters; (2) removing reads containing more than 10% unknown nucleotides (N); (3), and removing low quality reads containing more than 50% of low quality (*Q* ≤ 20) bases. The short reads alignment tool Bowtie2 (17) (version 2.2.8) was used for mapping the reads to ribosome RNA (rRNA) database. The rRNA mapped reads were then excluded, and the remaining reads were further assembled for transcriptome analysis. An index of the reference genome (GRCm38.p6) was built, and paired-end clean reads were mapped to the reference genome using HISAT2 (18) (version 2.1.0) with “rna-strandness RF” and other parameters set as a default. Transcript abundance was quantified by StringTie (19, 20) (version 1.3.4), using a reference-based approach. For each transcription region, an FPKM (fragment per kilo base of transcript per million mapped reads) value was calculated to quantify its expression abundance and variations.

### Sequencing Data Analyses

All sequencing raw data were deposited at Sequence Read Archive (NCBI SRA database) with the submission ID SRP278673. Differentially expressed transcripts (DEGs) were performed by DESeq2 (21) software between two different groups and by edgeR (22) between two samples. We considered mRNA with a fold change ≥2 and a false discovery rate (FDR) <0.05 in comparison as significant DEGs. Differentially expressed coding RNAs were then subjected to enrichment analysis of GO functions and KEGG pathways. All DEGs were mapped to GO terms in the Gene Ontology database (http://www.geneontology.org/). GO enrichment analysis provided all GO terms that were significantly enriched in DEGs, comparing them to the genome background and filtering the DEGs that corresponded to biological functions. The calculated *p*-value was analyzed with FDR Correction, using FDR ≤ 0.05 as a threshold. The GO terms that fulfilled this condition were defined as significantly enriched GO terms in DEGs. This analysis was able to uncover the main biological functions of DEGs. Pathway enrichment analysis using the KEGG database (23) identified significantly enriched metabolic or signal transduction pathways in DEGs. The Protein-Protein interaction (PPI) network was outlined using String v10 (24), which indicated genes as nodes and interaction as lines in the network. The network file was visualized using Cytoscape (v3.7.1) (25) software to present a core and hub gene biological interaction.

### qRT-PCR

Isolated RNAs were also subjected to qRT-PCR validation. RNAs were reverse-transcribed to cDNA by using a Prime-Script RT reagent Kit (TaKaRa, Dalian, China). Real-time PCR was performed using Light Cycler 480 II equipment (Roche Diagnostics, Basel, Switzerland). The primers used in this study are listed in Table 1. The reliability of primer sets and the quality of qRT-PCR experiments were validated by a single peak melt curve representing a single PCR product. The expression levels of target mRNAs were calculated by using the 2-ΔΔCt method by normalizing the expression vs. that of Gapdh.

**Table 1:** Primers of quantitative real-time PCR.

**Primer names**	**Forward (5**′**-3**′**)**	**Reverse (5**′**-3**′**)**
Runx2	CCTTCAAGGTTGTAGCCCTC	GGAGTAGTTCTCATCATTCCCG
Agtr2	TCCTGGGATTCACCAACAGC	CTCTCTTGCCTTGGAGCCAA
Itga2	TTCAGAGCAGAGTTTAGACCTG	AACACTTCTGCACTTCGTTTAC
Runx1	CCATCACCGTCTTTACAAATCC	ATCATCTAGTTTCTGCCGATGT
Itga8	CCCGATGGTGACTGGAACAA	TACACGCTCGAAGTTGCTGT
Agt	ATGAACTTGCCACTGGAGGG	GATGCTGTTGTCCACCCAGA
Gapdh	GGTTGTCTCCTGCGACTTCA	TGGTCCAGGGTTTCTTACTCC

### Statistical Analysis

The data were analyzed using SPSS, version 20.0 (SPSS, Inc. Chicago, IL). Quantitative data were presented as the mean ± SEM (standard error of the mean). The statistical significance between two experimental groups was analyzed using an unpaired two-sided *t*-test and *P* < 0.05 was considered as statistically significant.

## Results

### Increased Ascending Aortic Pressure and Remodeling of the Aortic Wall in the TAC Model

**Figure 1:**
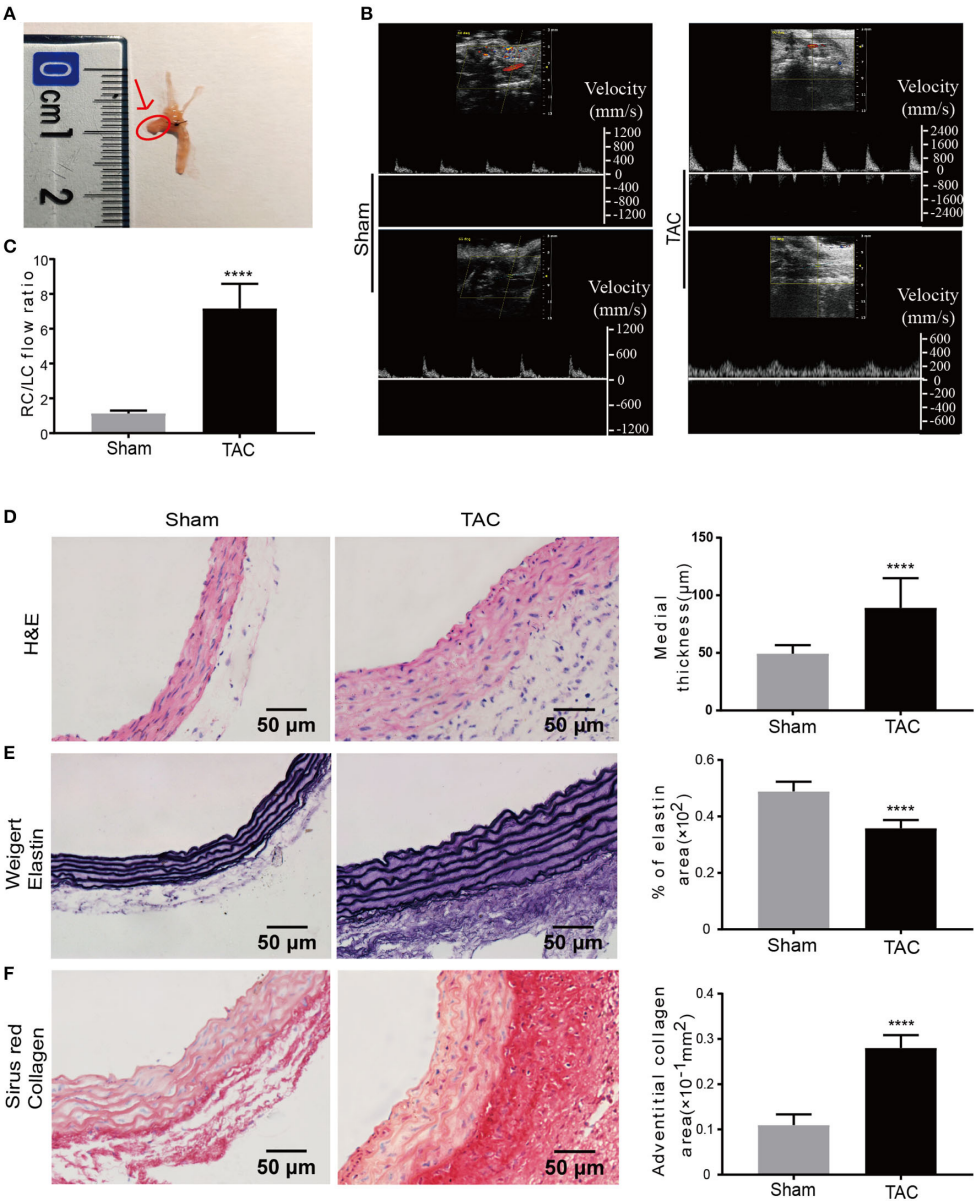
Increased blood pressure and remodeling of the aortic wall in established TAC model. **(A)** TAC was constructed by ligation between the brachiocephalic trunk and LCCA. Tissues used for all analysis are indicated by the arrow and the red circle; **(B)** Blood flow velocity measurement at RCCA and LCCA of the sham or TAC groups, respectively; **(C)** Right carotid /left carotid (RC/LC) flow ratio of TAC and sham group; **(D–F)** Representative images and quantification analysis of hematoxylin and eosin (H&E) staining **(D)**, Weigert's staining **(E)**, and Sirius red staining **(F)**; *n* = 6 per group. Original magnification: ×400. ****P < 0.0001.

Eight-week-old C57BL/6J mice were used to establish the TAC model by ligating between the brachiocephalic trunk and the left common carotid artery (LCCA) (Figure 1A). The blood flow velocity of the aortic ligated site (Supplementary Figure 1A), right common carotid artery (RCCA) (upper panel of Figure 1B), and LCCA (lower panel of Figure 1B) were determined by ultrasound 1 week after surgery. The blood flow velocity at the aortic ligation site of the TAC group was ~2.9 times higher than that of the sham group (Supplementary Figure 1B), indicating increased aortic pressure in the TAC group. TAC with RC/LC of 5–10 and the Sham ~ 1 (Figure 1C) were included in subsequent experiments. Two weeks after the operation, the diameter of the aortic ligament near the proximal end was measured by ultrasound (Supplementary Figure 1C), and the TAC group was 0.19 mm wider than the sham group on average (Supplementary Figure 1D).

Before sacrificing the mice at 2 weeks, they were subjected to an invasive catheter to detect pressure of left ventricle to reflect the pressure of the proximal end of ascending aorta. The results indicated that Left ventricular systolic pressure (LVSP) in the TAC group was significantly higher than the sham group (Supplementary Figures 2A,B). The maximum rate of change of the left ventricular pressure (dp/dt max) (Supplementary Figure 2C) and contractility (Supplementary Figure 2D) were also increased, suggesting enhanced systolic function of the heart in the TAC group., However, the indicators for diastolic function such as the left ventricular end-diastolic pressure (LVEDP) (Supplementary Figure 2B), minimum rate of change of the left ventricular pressure (dp/dt min) (Supplementary Figure 2C) and exponential time constant of relaxation (τ) (Supplementary Figure 2E) had no significant changes, suggesting no significant change in diastolic function in TAC treated mice.

The ascending aorta in the TAC group was significantly thickened when compared to the sham group 2 weeks after the TAC procedure, no aneurysms and thrombi were detected with microscopic examination. Morphological analysis revealed that the ascending aorta median thickness was significantly increased in the TAC group as much as 80.65% as compared to the sham group (Figure 1D). To further illustrate the remodeling of the aorta by TAC, Weigert's staining was performed to determine the elastic fibers of the media in TAC treated mice's ascending aorta (Figure 1E), indicating decreased compliance of the media in TAC group. Sirius red staining showed that collagen was dramatically accumulated in the ascending aorta of the surgery group, especially in the adventitia. The adventitia collagen deposition area was about 2.55 times larger than that of the sham group (Figure 1F). Of note, these histological changes induced by TAC are similar to those phenotypes observed in the ascending aorta with AngII infusion (26, 27) and in spontaneously hypertensive rats according to our previous study (28).

### Transcriptional Changes Identified by RNA-Seq in TAC Models

**Figure 2:**
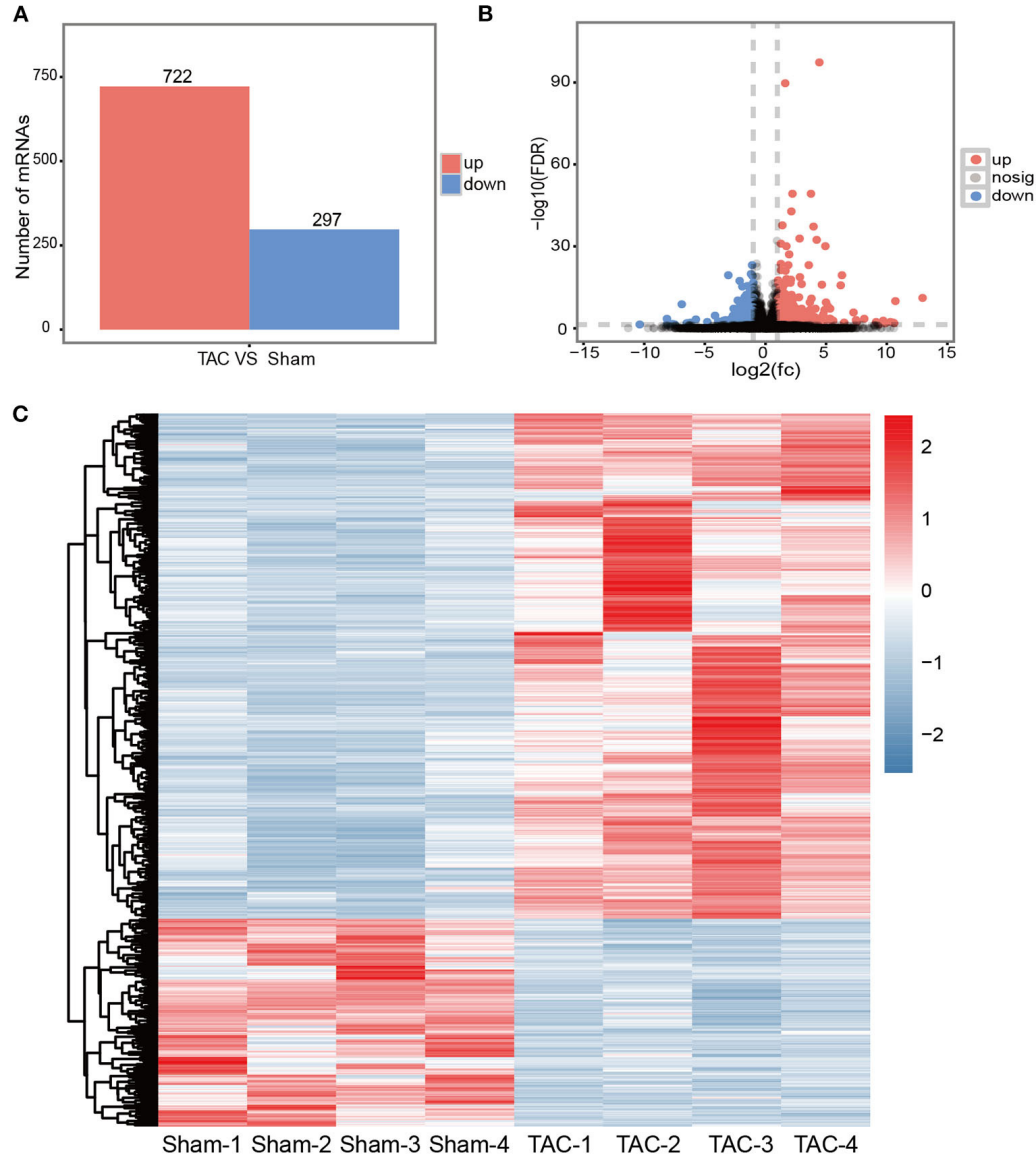
Transcriptional changes in TAC models. **(A)** An overview of DEGs in TAC models; **(B)** Volcano plot illustrating DEGs between the TAC and sham groups. Up-regulated mRNAs are labeled in red, down-regulated in blue, and the rest in gray; **(C)** Hierarchically clustered heat map illustrating DEGs between TAC and sham groups, fold change >2.0, *P* < 0.05 and FDR < 0.05, *n* = 4 per group.

The RNA-Seq was performed with ascending aortas dissected from TAC or sham control mice, respectively. Using well-established criterion including fold change> 2.0, *P* < 0.05 and FDR < 0.05, a total of 1,019 differentially expressed mRNAs were identified. Among them, 722 mRNAs were up-regulated and 297 were down-regulated in the TAC group (Figure 2A). The detailed information of DE mRNAs was listed in Additional file 1. A Volcano map was built with significantly differentially expressed genes (DEGs) between TAC and sham groups (Figure 2B), genes involved in immune response or ECM turnover, such as *Scube1, Scube2, C3ar1,C5ar2, Tlr6, Tlr7 and Col11a1,Col1a2, Itga11*, were highlighted. Interestingly, it is reported that Scube1is expressed in both mouse and human endothelial cells and over-expressed in the plasma of essential hypertension patients (29, 30), making it as a potential biomarker for hypertension diagnosis. Hierarchical clustering showed TAC and sham control were well-distinguished by differently expressed mRNA, with all the subjects correctly classified (Figure 2C). PCA analysis also revealed distinct expression profile of DEGs between the TAC and sham groups (Supplementary Figure 3).

qRT-PCR was performed to validate RNA deep sequencing results. A total of 6 mRNAs (*Runx2, Agtr2, Itga2, Runx1, Itga8, Ag*t) were randomly selected for validation. The qRT-PCR results showed that the expression patterns of these selected mRNAs were perfectly in consistent with the RNA deep sequencing data (Supplementary Figure 4).

### Functional Enrichment Assay of Transcripts in TAC Models

**Figure 3:**
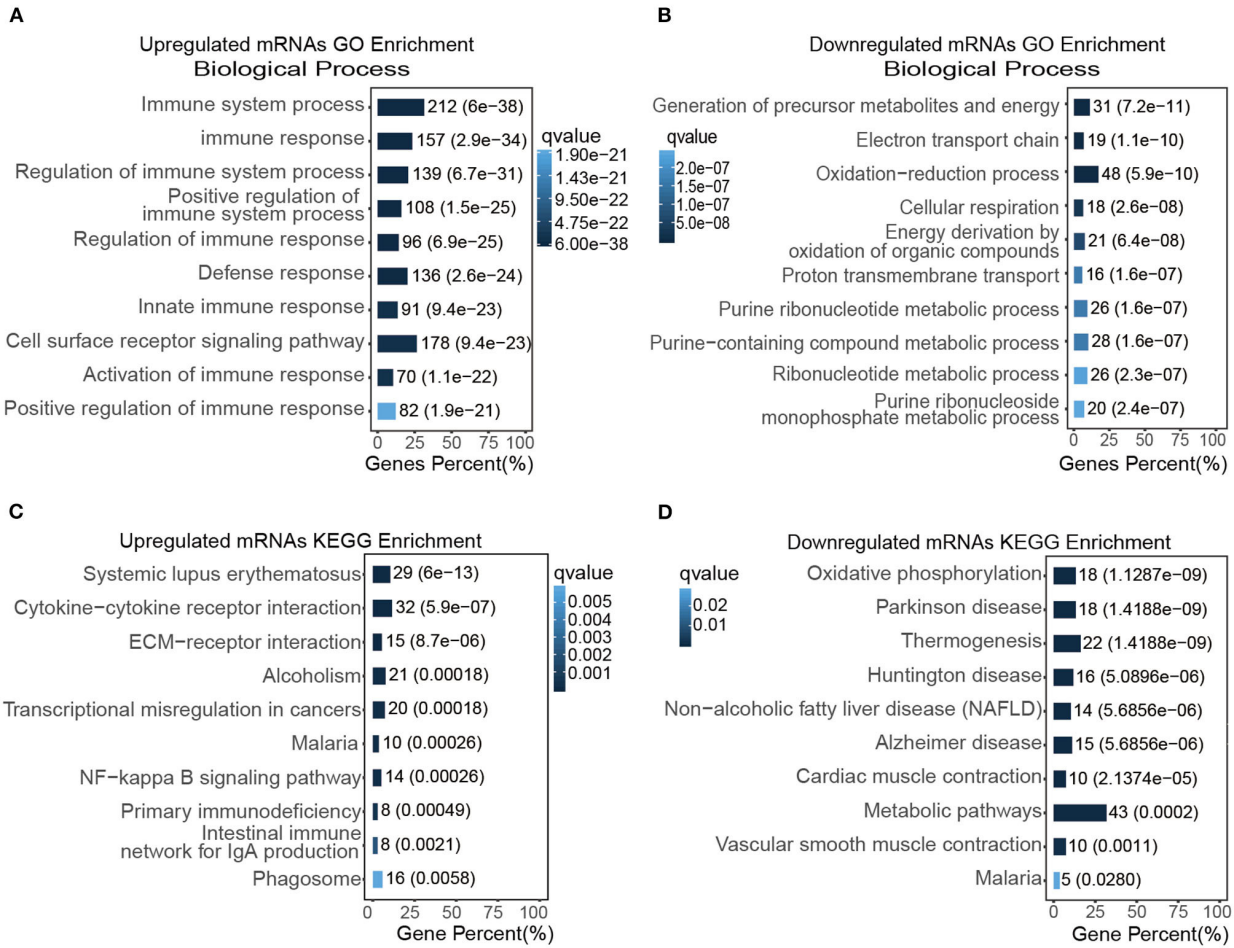
Functional enrichment assay of transcripts in the TAC model. **(A,B)** The top GO enriched biological functions of up **(A)** and down-regulated DEGs **(B)**, respectively; **(C,D)** the top KEGG enrichment items of up **(C)** and down-regulated DEGs **(D)**, respectively.

We then analyzed the molecular functions of differentially expressed mRNAs using GO and KEGG databases. The top GO enrichment terms of the biological process were immune system process and cell surface receptor signaling pathway (Figure 3A); the top enrichment terms for cellular component were extracellular region, extracellular matrix, and collagen-containing extracellular matrix (Supplementary Figure 5A); the top enrichment terms for molecular function were extracellular matrix structural constituent and protein binding (Supplementary Figure 5C). In addition, the inflammatory response, lymphocyte activation, and regulation of response to stimulus, were also up-regulated in the TAC group as revealed by GO enrichment analysis. For those down-regulated transcripts, the most enriched term of the biological process was the oxidation-reduction process (Figure 3B); the mitochondrial part and mitochondrion were the most enriched terms for cellular component (Supplementary Figure 5B); and the oxido-reductase activity as well as cofactor binding were the top enriched terms for molecular function (Supplementary Figure 5D).

To further analyze the DEGs, KEGG pathway enrichment was used to analyze those up-regulated transcripts (Figure 3C) or down-regulated transcripts (Figure 3D), respectively. Our result suggested that cytokine-cytokine receptor interaction, ECM-receptor interaction, NF-kappa B signaling pathway were highly activated and enriched in TAC group, in contrast, oxidative phosphorylation, cardiac muscle contraction, and vascular smooth muscle contraction were inhibited in the TAC group. Intriguingly, the p53 signaling pathway was also up-regulated in KEGG enrichment analysis. These results collectively indicated that immune response, stress/stimulus-related processes played a vital role in pressure overload induced ascending aorta remodeling.

### The Interactions Network of Most Significant DEGs in the TAC Model

**Figure 4:**
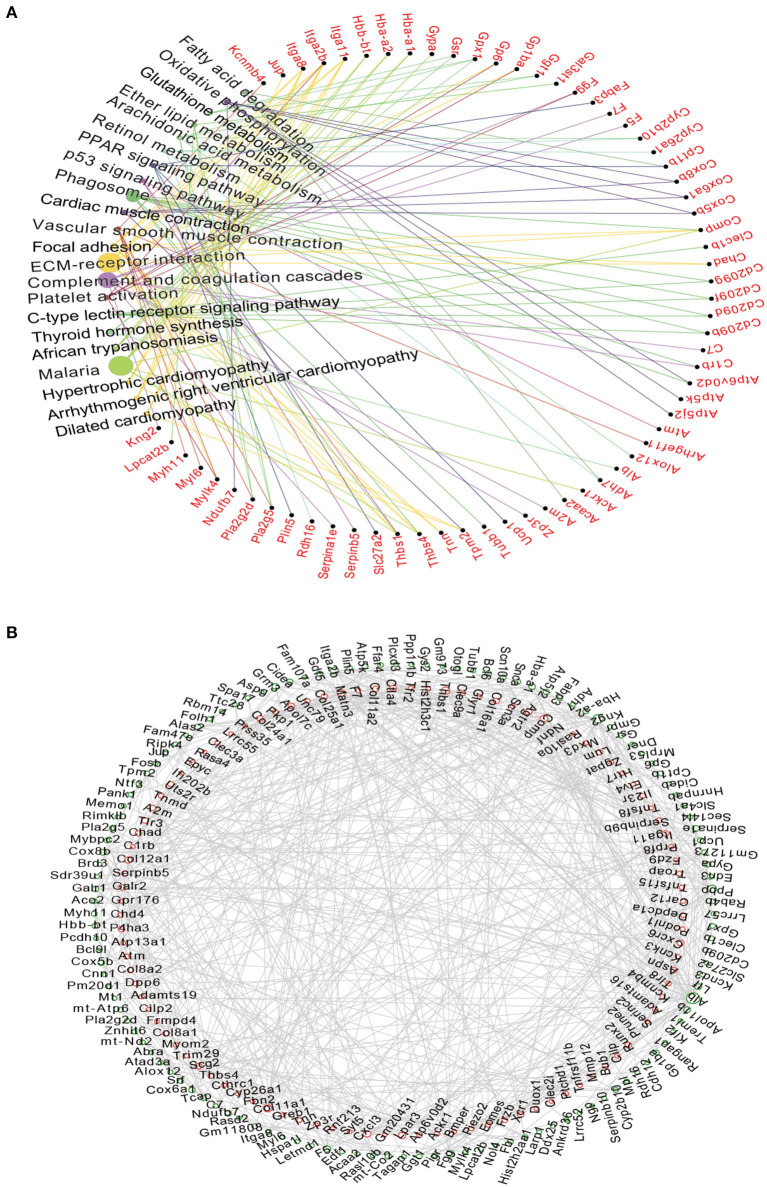
The Network analysis of DEGs and predicted PPI networks in the TAC group. **(A)** KEGG analysis suggested that differentially mRNAs were mainly targeted to the ECM-receptor interaction and Complement and coagulation cascades. Circle size represented degree; **(B)** The network showed the potential relationship between the top 200 differentially up-regulated and down-regulated expressed mRNAs, Up-regulated mRNAs are displayed in the red circle, down-regulated in the green circle.

The KEGG analysis was further performed in the top 200 up-regulated and top 200 down-regulated DEGs, and the results showed that the major enrichment pathways were ECM-receptor interactions (e.g., *Itga2b, Tnn, Thbs1*, and *Itga11*), complement and coagulation cascades (e.g., *Fgg, C7, A2m*, and *Gpx1*) and vascular smooth muscle contraction (e.g., *Pla2g5, Myh11, Myl6, Kcnmb4*, and *Mylk4*) (Figure 4A). We then used the String database to establish a protein-protein interaction (PPI) network using top 200 up-regulated and top 200 down-regulated DEGs (Figure 4B). Accordingly, those up-regulated DEGs such as *Cxcr6, Cxcl3, Tlr3, Thbs1, Col11a1, Angptl1, Mmp12, Itga11, Bmper, Col8a1*, and *Piezo2*, and those down-regulated DEGs like *Alb, Kng2, Itga2b, Cox5b, Cox6a1*, and *Fgg* are promising targets for further investigation (Figure 4B).

### Validation of DEGs Revealed by RNA-Seq

**Figure 5:**
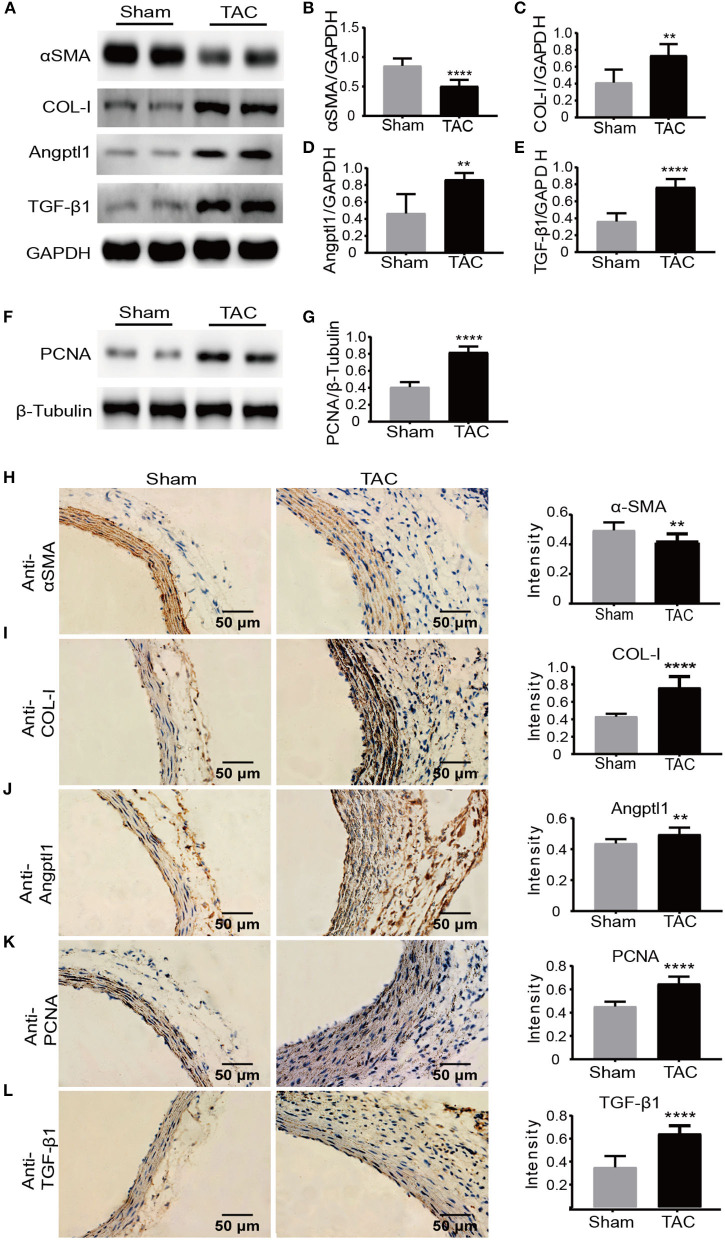
Protein level validation of DEGs revealed by RNA-seq. **(A–G)** Western blot analysis of α-SMA, COL-I, Angptl1, TGF-β1, and PCNA in the TAC and sham groups. GAPDH and β-Tubulin were used as endogenous loading controls. Lysates were prepared from ascending aortas of sham-operated or TAC mice, *n* = 5 per group. The experiments were repeated 3 times independently; **(H–L)** Immunohistochemistry and quantification analysis of α-SMA, COL-I, Angptl1, TGF-β1, and PCNA in ascending aortas derived from TAC or sham controlled mice. *n* = 6 per group. Original magnification: ×400. Scale bars: 50 μm. **P < 0.01, ****P < 0.0001.

To validate our findings of those transcriptional changed DEGs, we then analyzed the protein expression level using multiple approaches. First, we determined the cell proliferation and immune response *in situ* by staining with PCNA and TGF-β1. In line with the previous finding, we observed a significantly increased expression of PCNA and TGF-β1 in adventitia in the TAC model (Supplementary Figure 6). Intriguingly, α-smooth muscle actin (α-SMA), a surface marker of contractile smooth muscle cells, was significantly decreased in TAC model (Figures 5A,B); in contrast, Collagen I (COL-I) (Figures 5A,C), a Angptl1 (Figures 5A,D) TGF-β1 (Figures 5A,E) and PCNA (Figures 5F,G) were highly expressed in the ascending aorta where remodeling occurred in TAC model as indicated by immuno-blot analysis. Immunohistochemistry analysis with ascending aorta tissues also showed that down-regulation of α-SMA (Figure 5H), but up-regulation of COL-1 (Figure 5I), TGF-β1 (Figure 5L), Angptl1 (Figure 5J) and PCNA (Figure 5K) in TAC model as compared to sham surgery treated mice. These results collectively support previous RNA-seq data, which indicates that the immune response and ECM-receptor interaction played a crucial role during high blood pressure induced aortic remodeling.

## Discussion

It is widely accepted that the main pathological outcome of hypertension is vascular remodeling. In principle, when hemodynamic changes occur, the shear force decreases, leading to the activation of vascular endothelial cell signaling transduction. This then induces a cascade of downstream responses, such as medial smooth muscle cells and adventitial fibroblasts morphological transformation, infiltration of early inflammatory cells by adventitial cells (31–33). During this process, smooth muscle cells are switched from contractile types to synthetic types, which is the iconic event of vascular remodeling (34–36). However, the underlying molecular mechanism of how hypertension induces vascular remodeling and ultimately organ damage remains unclear. Therefore, the early intervention of this process, by targeting those key regulators or pathways, serves as a promising strategy to delay or prevent hypertension associated with pathological consequences.

Recent studies have shown that the TAC model is an efficient system to recapitulate the infiltration of adventitial macrophages and conversion of adventitial fibroblasts to myofibroblasts, with significant vascular remodeling (37, 38). In response to vascular pressure or injury, vascular adventitia fibroblasts are usually the first sensor to sense hemodynamic changes (39), but how the fibroblasts that are located in the outer membrane transform into myofibroblasts is not fully understood. It is reported that adventitia fibroblasts undergo phenotypic transformation upon sensing vascular injury or blood flow stress and participate in vascular remodeling (38). Activated myofibroblasts not only synthesize large amounts of ECM but also affect the phenotypic transformation, proliferation, apoptosis, and migration of VSMC. When treated with Ang-II, VSMCs proliferate and migrate, and the expression of α-SMA, a marker for the contractile phenotype of VSMC and myofibroblasts, was decreased (40). A previous study showed that in the TAC model, the expression of α-SMA in the adventitia was increased, but no significant difference was detected in the media 2 weeks after TAC surgery (13). Paradoxically, in our study, the RNA-seq data, protein level analysis as well as *in situ* immunohistochemistry indicated that α-SMA were down-regulated in the ascending aorta in the TAC group as compared to the sham group. It is speculated that the phenotypic transformation of media smooth muscle cells and adventitia fibroblasts might contribute to the different expression patterns of α-SMA in the media and adventitia of remodeling arteries.

In our study, we successfully established a TAC model with morphological and immunohistological validation of vascular tissues, and these tissues were directly subjected to RNA sequencing analysis. Our results showed that upon vascular remodeling, the genes related to extracellular matrix transformation and smooth muscle cell contraction were significantly changed, not surprisingly, inflammation-related genes were also significantly enriched in our analysis, which was consistent with the classical theory that inflammation played an essential role in hypertension-induced vascular remodeling (10) and suggested that anti-inflammation might be a useful strategy to inhibit hypertension associated pathological consequences. Nevertheless, it would be more convincing to use other hypertension or vascular remodeling models to investigate whether our findings were representative. Ultimately, clinical studies with early stage hypertension patients are required to validate these findings.

Among those differentially expressed genes, we observed that many of them were involved in stress/stimulus/immune pathways. For instance, a classical immune response regulator, TGF-β1 was significantly up-regulated at both transcriptional and protein levels. High TGF-β1 expression usually indicated an immune-suppressive micro-environment, which suggested that the tissues of vascular remodeling might lack proper immune surveillance, e.g., increased infiltration of macrophages or regulatory T cells, suggesting that targeting the immune system using small molecular chemicals or mono-antibody might also be useful to treat hypertension-related complications at an early stage. Whether this method could ameliorate vascular remodeling in the TAC model requires further investigation.

The main shortcomings of our study were the age factor and the inadequate time frame. Hypertension is rarely be observed in the young mice we used for the current study and it is also known that mice have an age-related response to cardiac pathologies (41); furthermore, the time frame to perform the TAC study might have been too short to reflect the chronic changes that will occur within the remodeled vessels, thus, a time-dependent study might be necessary to properly mimic clinical phenotype progression. On the other hand, most of the current available classical animal models for the study of hypertension were mainly conducted in aged mice over a longer time-frame. Therefore, it is unfeasible to investigate early pathological events and evaluate whether early interventions would delay or prevent hypertension development. Importantly, the TAC model used for the current study faithfully mimics those early hypertensive pathological changes, e.g., increased blood pressure and aortic remodeling as early as 2 weeks after the ligation surgery. Moreover, a big advantage of this model was that it could be used to quickly estimate if clinically available drugs/drug candidates/herbs/small interfering RNAs could ameliorate such pathological changes, providing a rapid and efficient platform to screen potential therapeutic strategies which can be further validated with other classical hypertension models or clinical trials.

Taken together, we identified a different expressed mRNAs profile, which was associated with the progression of aortic remodeling in the TAC model. These differentially expressed genes might function and interact mutually as a network to mediate hypertension-induced vascular remodeling. Our research suggests that manipulating inflammation and targeting the immune system are potential therapeutic strategies to prevent aortic remodeling induced by blood pressure overload.

## Data Availability Statement

All sequencing raw data were submitted and deposited at Sequence Read Archive (NCBI SRA database) with the submission ID PRJNA659049.

## Ethics Statement

The animal study was reviewed and approved by Institutional Animal Care and Use Committee of Southern Medical University.

## Author Contributions

LZ and JX contributed to the experimental design and manuscript draft. XZ and TF contributed to the experimental design and implementation. X-XZ, BS, QZ, and YC contributed to the data analysis and manuscript draft. HL, FZ, TW, DC, and XL contributed to the experimental implementation and data analysis. All authors reviewed and approved the final manuscript.

## Conflict of Interest

The authors declare that the research was conducted in the absence of any commercial or financial relationships that could be construed as a potential conflict of interest.
