# Supplementary material for: Identification of Transcriptional Variation in Aortic Remodeling Using a Murine Transverse Aortic Constriction (TAC) Model
Source: Front Cardiovasc Med. 2020 Nov 12;7:581362. doi: 10.3389/fcvm.2020.581362 (PMC7693635; doi:10.3389/fcvm.2020.581362)
Supplement: Supplementary file 1 [file Data_Sheet_1.DOCX]

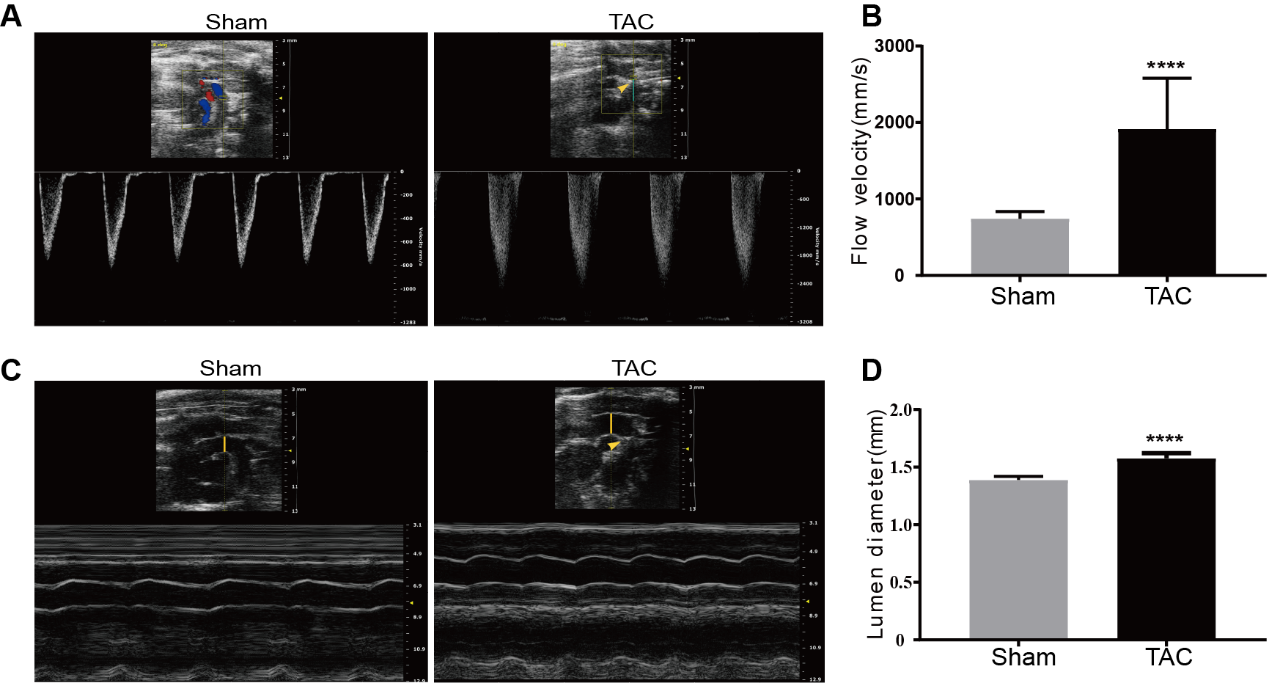


**Figure S1.** Echocardiographic analysis of aortic arch of TAC-mediated aortic remodeling. **(A)** Blood flow velocity measurement at aortic arch banding sites of the Sham or TAC groups; **(B)** Quantification of blood flow velocity in the Sham and TAC groups; **(C)** Lumen diameter determination of the aortic ligament near the proximal end; **(D)** Quantification analysis of the Lumen diameter of Sham or TAC groups. ****, P < 0.0001.


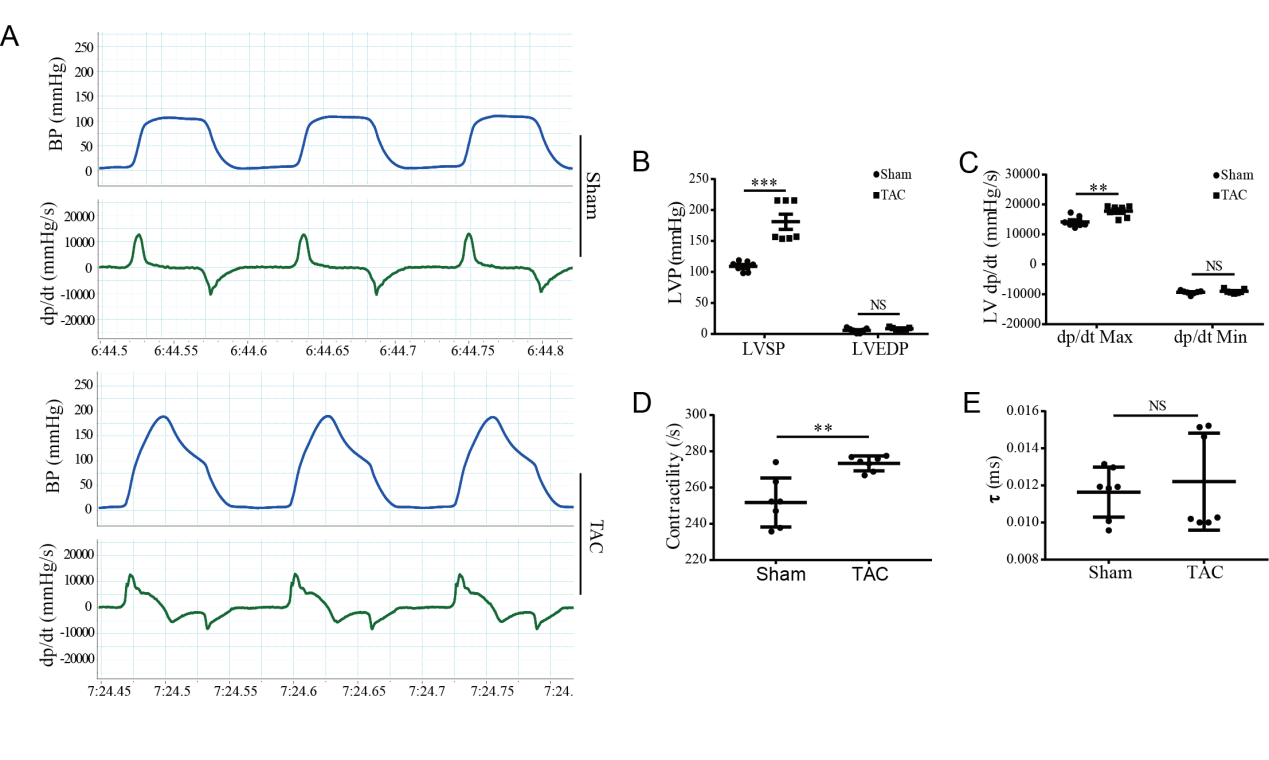


**Figure S2**. **Evaluation of invasive hemodynamic parameters in the mice 2 weeks after Sham surgery or TAC.**

(A) Representative recordings of left ventricular pressure and the change of left ventricular pressure in Sham and TAC group; (B) Determination of left ventricular systolic pressure (LVSP) and left ventricular end-diastolic pressure (LVEDP) in Sham and TAC group; (C) Maximum rate of change of the left ventricular pressure (dp/dt max) and minimum rate of change of the left ventricular pressure (dp/dt min); (D) Left ventricular contractility determination in Sham and TAC group; (E) Exponential time constant of relaxation (τ); n = 3 per group, **, P < 0.01; ***, P < 0.001.


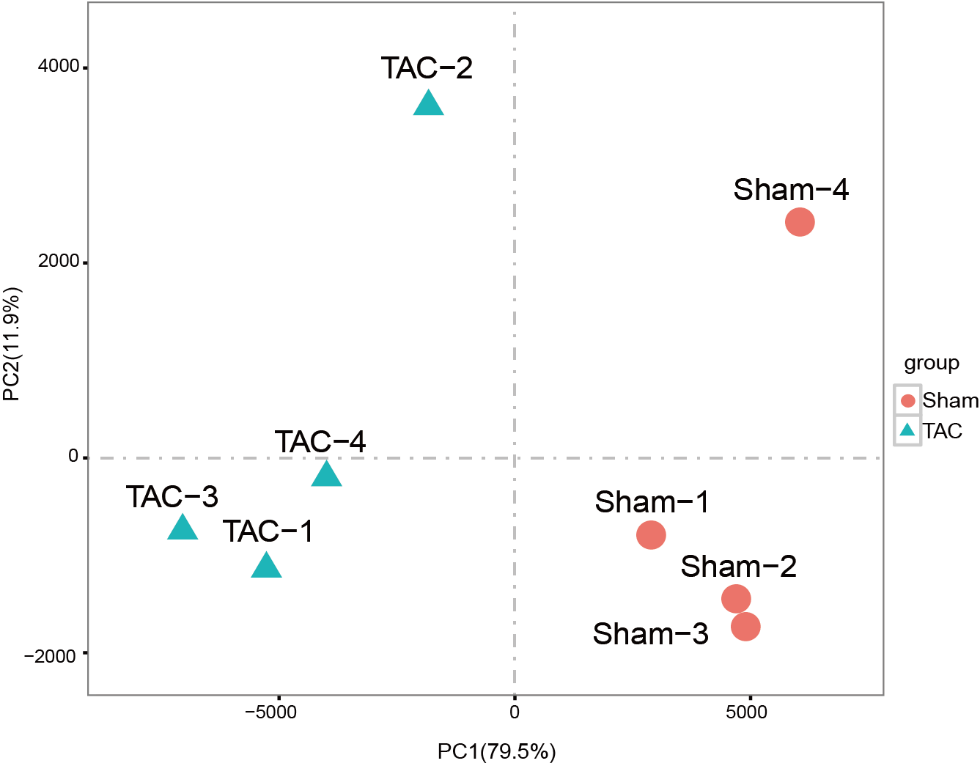


**Figure S3.** PCA assay of DEGs in the TAC model. The PCA analysis showed that DEGs in the Sham and TAC group were categorized into two different subgroups based on RNA-seq data analysis.


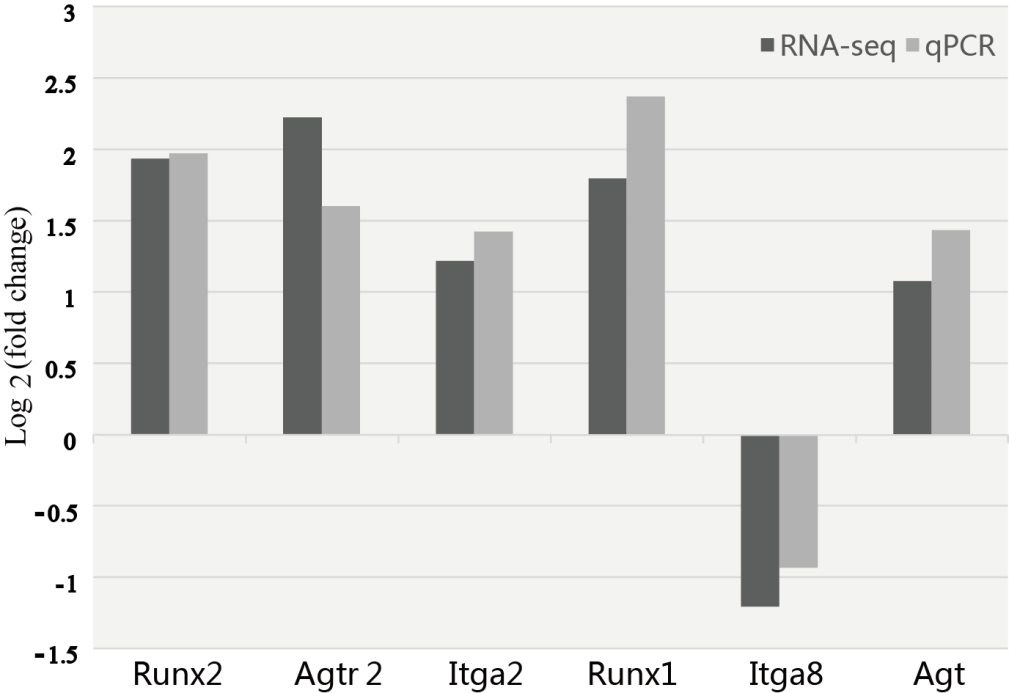


**Figure S4**. **Validation and comparison of selected genes' expressions by qRT-PCR and RNA-seq.**

qRT-PCR analysis was performed to determine the expression of *Runx2, Agtr2, Itga2, Runx1, Itga8, and Agt* in the TAC group, the value of the Sham group were set as 1. The individual gene's expression was also re-analyzed by RNA-seq data (Grey).


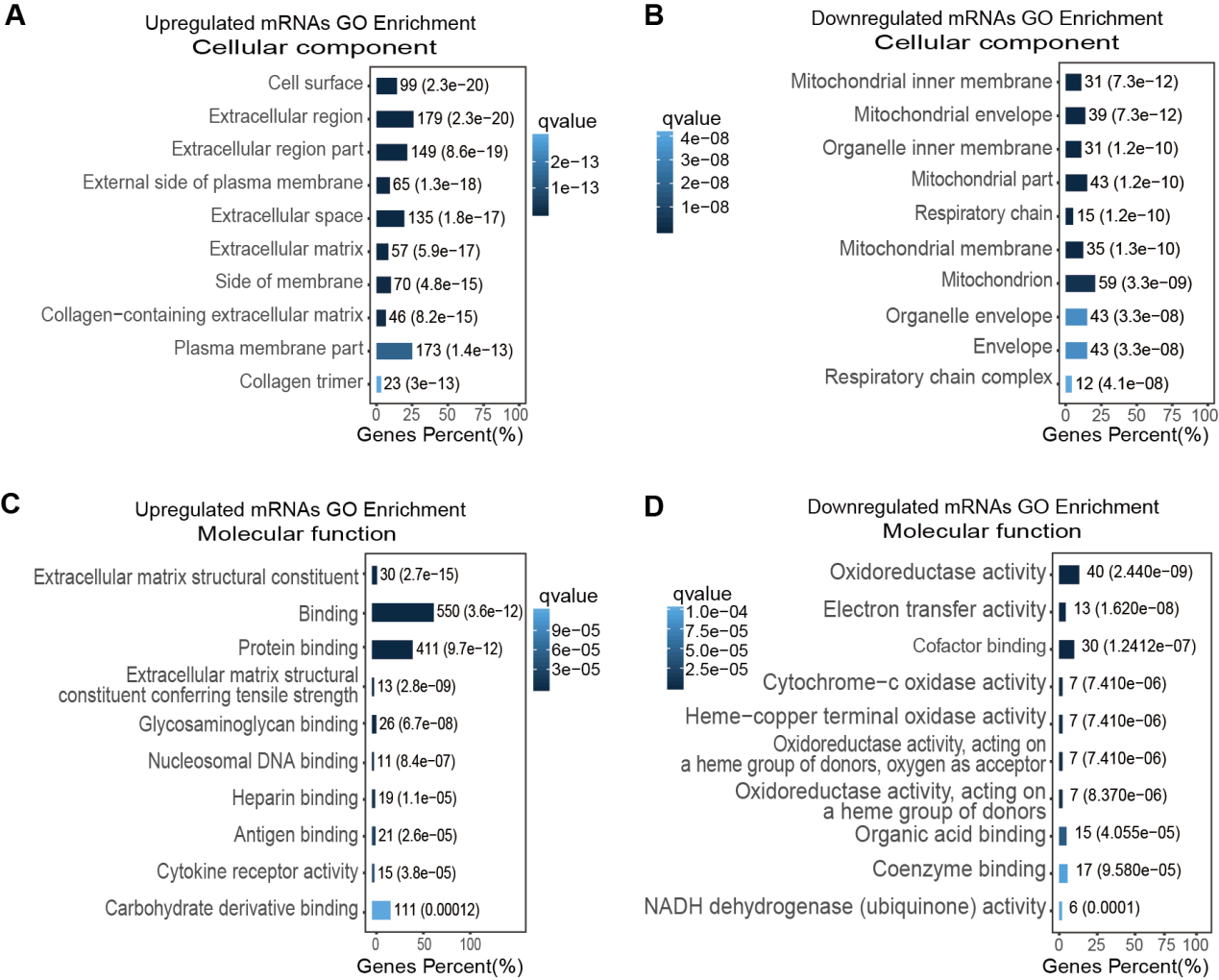


**Figure S5. GO enrichment assay of DEGs in the TAC model. The top GO terms are enriched in cellular component (A, B) and molecular functions (C, D).**


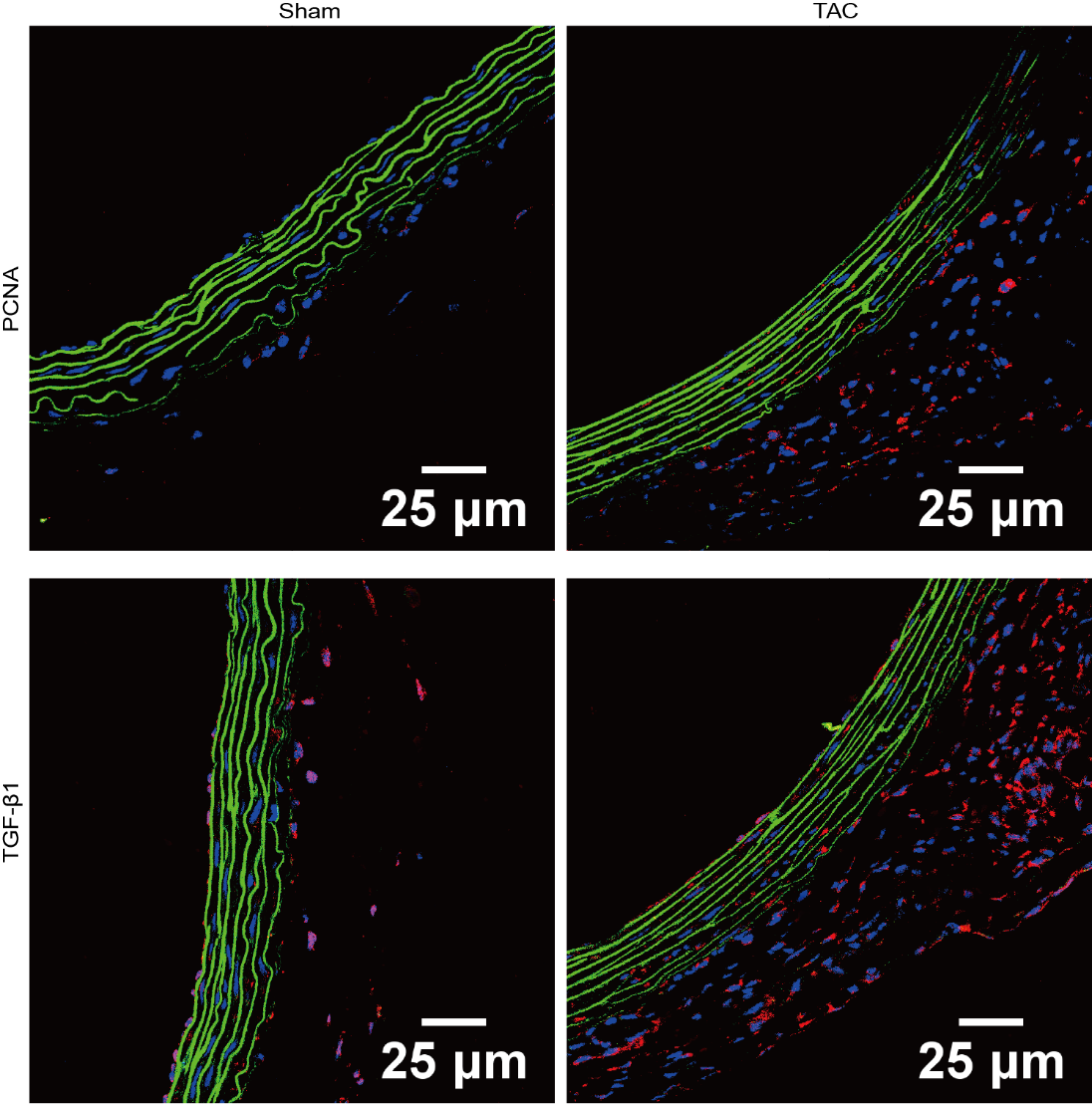


**Figure S6. Immunofluorescence analysis of TAC-mediated aortic remodeling.** Immunofluorescence staining for PCNA and TGF-β1. Nuclei were stained with DAPI (blue). Elastic lamellae of the media are green (autoﬂuorescence). Positive staining of PCNA and TGF-β1 is red. n=6 per group. Original magnification: ×400.
